# Combination of AQP1 and β-catenin expression is an independent prognosis factor in astrocytoma patients

**DOI:** 10.18632/oncotarget.19562

**Published:** 2017-07-26

**Authors:** Huikun Zhang, Fengxia Qin, Limin Yang, Jia He, Xiaoli Liu, Ying Shao, Zhifang Guo, Ming Zhang, Wenliang Li, Li Fu, Feng Gu, Yongjie Ma

**Affiliations:** ^1^ Department of Breast Cancer Pathology and Research Laboratory, Tianjin Medical University Cancer Institute and Hospital, Tianjin, China; ^2^ Department of Tumor Cell Biology, Tianjin Medical University Cancer Institute and Hospital, Tianjin, China; ^3^ Department of Neurosurgery, Tianjin Medical University Cancer Institute and Hospital, Tianjin, China; ^4^ National Clinical Research Center for Cancer, Tianjin, China; ^5^ Tianjin’s Clinical Research Center for Cancer, Tianjin, China; ^6^ Key Laboratory of Cancer Prevention and Therapy, Tianjin, China; ^7^ Department of Epidemiology and Biostatistics, Biomedical Institute, University of Georgia, Athens, GA, USA

**Keywords:** aquaporin1, β-catenin, prognosis, astrocytoma, combination

## Abstract

Previous research usually focused on single protein or gene in tumor development, actually highly heterogeneous nature and different signaling pathways largely contribute to tumor progression and tumor patients’ outcomes. Therefore, using combinatorial biomarkers to evaluate the prognostic features and guide management is gradually accepted and urgently needed. β-catenin is a well-known crucial factor in astrocytoma progression and it is involved in aquaporin1 (AQP1) mediated cell migration. In this study, we revealed the function of AQP1 in astrocytoma progression and provided the first clinical evidence that AQP1 expression was positively correlated with β-catenin. Furthermore, we proved the functional role of AQP1/β-catenin pathway in astrocytoma progression. More importantly, we discovered that combination of AQP1 and β-catenin expression was an independent prognosis factor for astrocytoma patients and it was a better survival predictor than either AQP1 or β-catenin alone. In conclusion, our study provided a novel more precise prognostication for predicting astrocytoma prognosis based on combinatorial analysis of AQP1 and β-catenin expression.

## INTRODUCTION

Astrocytomas are the most common primary intracranial tumors in adults [1]. Despite recent treatment advances, patients’ prognosis remains poor; a majority of glioblastoma patients succumb to the disease within 2 years of diagnosis [2]. Previous research usually focused on single protein or gene, which has been implicated in prediction of astrocytoma prognosis on a certain stage [3, 4]. Actually, highly heterogeneous nature and mechanisms of interaction such as crosstalk between different signaling pathways are likely to largely contribute to tumor development and distinct outcomes of patients [5]. Therefore, using combinatorial immunohistochemical markers to evaluate the prognostic features and guide management is gradually accepted and urgently needed in astrocytoma clinical practice [6–8].

Aberrant activation of Wnt/β-catenin signaling has been described in a range of malignancies including astrocytoma [9, 10]. As a key molecule in Wnt signaling pathway, β-catenin was important in both proliferation and metastasis of tumor cells. Down-regulation of β-catenin inhibited cell proliferation and invasive abilities, and induced apoptotic cell death [11, 12]. On the contrary, overactivation of canonical Wnt/β-catenin signaling led to unregulated expression of metastasis-associated genes (such as MMP2 and MMP9) and epithelial-to-mesenchymal transition activators (such as ZEB1, Twist and Snail) and increased *in vitro* cell migration/invasion [13, 14].

Recently, β-catenin was revealed to be involved in aquaporin 1 (AQP1) mediated cell migration by *in vitro* studies [15–17]. AQP1 was not only a water channel but also a critical scaffold for plasma-membrane associated multiprotein-complex important for cytoskeleton build-up, adhesion and motility. AQP1 combined with β-catenin led to the re-organization of the cytoskeleton and cell migration [17]. Furthermore, both Li and Caterina’s groups reported that AQP1 could co-immunoprecipitate with β-catenin and overexpression of AQP1 up-regulated β-catenin expression [16, 18]. However, of note, the functional roles of AQP1 in astrocytoma progression are unclear due to limited research and a restriction of sample size. Furthermore, the association of AQP1 and β-catenin in clinic and the prognostic value of their combination have not been discovered up to now.

In the present study, we explored the relationship between AQP1 and β-catenin by clinical and cellular analyses and demonstrated for the first time that AQP1 expression was positively correlated with β-catenin in astrocytoma specimens. We also discovered the functional role of AQP1/β-catenin pathway in astrocytoma progression. More importantly, we discovered that combination of AQP1 and β-catenin expression was an independent prognosis factor for astrocytoma patients and it was a better survival predictor than either AQP1 or β-catenin alone. Patients with AQP1 low/β-catenin low had the best outcome while the survival of the other three subgroups (with high expression of one or both of them: AQP1 low/β-catenin high, AQP1 high/β-catenin low, and AQP1 high/β-catenin high) was similar. Either high expression of AQP1 or β-catenin indicated a poor outcome. In conclusion, our study provided a novel more precise prognostication for predicting astrocytoma prognosis based on combinatorial analysis of AQP1 and β-catenin expression.

## RESULTS

**Figure 1:**
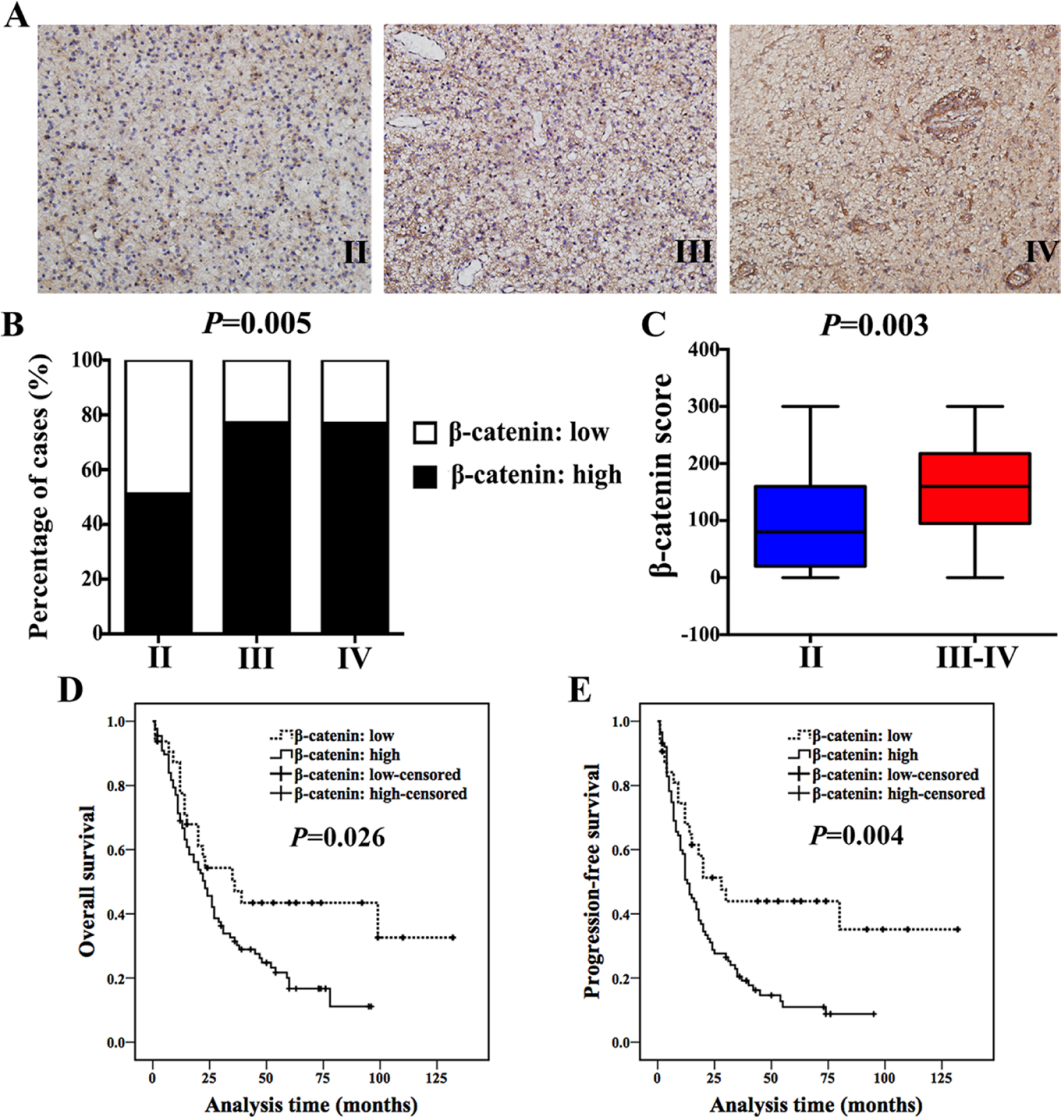
β-catenin expression increased with histological grade and inversely correlated with patients’ outcome (**A**) IHC staining of β-catenin in Grade II, III and IV astrocytoma specimens (magnification, 200×). (**B**) 51% (24/47) of Grade II patients exhibited high expression of β-catenin, while 77.1% (37/48) of Grade III cases and 76.9% (50/65) of Grade IV showed high expression of β-catenin (χ^2^ test, *P* = 0.005). (**C**) Ranges and median values of β-catenin score in astrocytoma specimens (Mann-Whitney *U* test, *P* = 0.003). (**D** and **E**) High expression of β-catenin indicated a shorter OS (D) and PFS (E) in astrocytoma patients (log-rank test).

In the current study, we examined the expression of β-catenin in a cohort of 160 astrocytoma specimens (Grade II–IV, Table 1) and explored its clinical relevance and prognostic significance at the first step. Patients’ clinicopathological information was shown in Table 1. We found that expression of β-catenin increased with histological grade (Figure 1A and 1B, Table 2). β-catenin expression in high-grade astrocytoma was much higher than that in low-grade astrocytoma (*P* = 0.003, Figure 1C). In addition, high expression of β-catenin indicated a poor prognosis of astrocytoma patients (Figure 1D and 1E). However, it was worth noting that β-catenin was not an independent prognostic factor in multivariate analysis, stratified by age, tumor size and histological grade (*P* = 0.133, Table 4).

**Table 1:** Demographic data of patients

Variable	Value
Age (years), mean (range)	49 (9–80)
Gender	
Male	95
Female	65
Histological grade	
Grade II	47
Grade III	48
Grade IV	65
Location	
Frontal	54
Temporal	47
Parietal	14
Occipital	7
Cerebellum	4
Callosum/ventricle/thalamus	12
More than one lobe	22
Tumor size (cm)	4.7 ± 1.6 (0.5–9.5)
Surgery	
Partial	28
Complete	132
Peritumoral edema	
Weak	35
Moderate	78
Strong	47
Family history	
Yes	6
No	154
Radiotherapy (post operation)	
Yes	72
No	88
Chemotherapy (post operation)	
Yes	103
No	57
Events^a^	
Death	88 (follow-up: 1–99 months; median: 15 months)
Censored	32 (follow-up: 2–132 months; median: 60 months)
Recurrence^a^	
Yes	94 (follow-up: 1–80 months; median: 12 months)
No	26 (follow-up: 2–132 months; median: 53 months)

^a^Some missing data.

**Table 2:** β-catenin expression and clinicopathological variables of astrocytoma

Pathological features	Cases	β-catenin score, *n* (%)			*r*_s_	*P* value^a^
		0-99	100-199	200-300		
Age^b^	160					0.542
Tumor size^c^	160					0.503
Gender					0.100	0.209
Male	95	31 (32.6)	42 (44.2)	22 (23.2)		
Female	65	18 (27.7)	25 (38.5)	22 (33.8)		
Histological grade					0.287	0.000
Grade II	47	23 (48.9)	19 (40.4)	5 (10.6)		
Grade III	48	11 (22.9)	24 (50.0)	13 (27.1)		
Grade IV	65	15 (23.1)	24 (36.9)	26 (40.0)		
Peritumoral edema					0.032	0.689
Weak	35	9 (25.7)	15 (42.9)	11 (31.4)		
Moderate	78	28 (35.9)	33 (42.3)	17 (21.8)		
Strong	47	12 (25.5)	19 (40.4)	16 (34.0)		
Location					0.096	0.192
Frontal	54	18 (33.3)	23 (42.6)	13 (24.1)		
Temporal	47	13 (27.6)	17 (36.2)	17 (36.2)		
Parietal	14	6 (42.8)	4 (28.6)	4 (28.6)		
Occipital	7	3 (42.9)	4 (57.1)	0 (0.0)		
Cerebrum	4	1 (25.0)	3 (75.0)	0 (0.0)		
Callosum/ventricle/thalamus	12	5 (41.7)	3 (25.0)	4 (33.3)		
More than one lobe	22	3 (13.6)	13 (59.1)	6 (27.3)		

^a^*P* values were calculated by Spearman’s Rank-Correlation test.

^b^Age: 49 (9–80) years, F = 0.619, *P* = 0.542 (ANOVA test).

^c^Tumor size: (4.7 ± 1.6) cm, F = 0.690, *P* = 0.503 (ANOVA test).

**Table 3:** AQP1 expression and clinicopathological variables of astrocytoma

Pathological features	Cases	AQP1 score, n (%)			*r*_s_	*P* value^a^
		0	1-4	5-9		
Age^b^	160					0.733
Tumor size^c^	160					0.587
Gender					−0.122	0.123
Male	95	5 (5.3)	21(22.1)	69 (72.6)		
Female	65	3 (4.6)	23 (35.4)	39 (60.0)		
Histological grade					0.163	0.039
Grade II	47	8 (17.0)	11 (23.4)	28 (59.6)		
Grade III	48	0 (0.0)	16 (33.3)	32 (66.7)		
Grade IV	65	0 (0.0)	17 (26.2)	48 (73.8)		
Peritumoral edema					−0.006	0.943
Weak	35	3 (8.6)	9 (25.7)	23 (65.7)		
Moderate	78	4 (5.1)	19 (24.4)	55 (70.5)		
Strong	47	1 (2.1)	16 (34.0)	30 (63.8)		
Location					−0.003	0.972
Frontal	54	3 (5.6)	16 (29.6)	35 (64.8)		
Temporal	47	1 (2.1)	11 (23.4)	35 (74.5)		
Parietal	14	1 (7.1)	5 (35.7)	8 (57.1)		
Occipital	7	2 (28.6)	0 (0.0)	5 (71.4)		
Cerebrum	4	0 (0.0)	1 (25.0)	3 (75.0)		
Callosum/ventricle/thalamus	12	1 (8.3)	3 (25.0)	8 (66.7)		
More than one lobe	22	0 (0.0)	8 (36.4)	14 (63.6)		

^a^*P* values were calculated by Spearman’s Rank-Correlation test.

^b^Age: 49 (9–80) years, F = 0.311, *P* = 0.733 (ANOVA test).

^c^Tumor size: (4.7 ± 1.6) cm, F = 0.534, *P* = 0.587 (ANOVA test).

**Table 4:** Univariate and multivariate analysis for overall survival (OS) analysis

Variables	Univariate			Multivariate			Multivariate		
	HR	95% CI	*P* value	HR	95% CI	*P* value	HR	95% CI	*P* value
Age	1.031	1.017–1.045	0.000	1.025	1.010–1.040	0.001	1.026	1.011–1.041	0.001
Gender (male/female)	0.949	0.620–1.455	0.812						
Tumor size (≤ 5 cm versus > 5 cm)	1.795	1.169–2.758	0.008	1.812	1.162–2.825	0.009	1.889	1.205–2.961	0.006
Histological grade (II versus III versus IV)	1.966	1.486–2.599	0.000	1.716	1.272–2.316	0.000	1.761	1.315–2.357	0.000
Peritumoral edema	1.249	0.951–1.641	0.110						
Surgical excision (complete versus partial)	0.697	0.399–1.216	0.204						
Radiotherapy (yes/no) (post operation)	0.725	0.476–1.105	0.135						
Chemotherapy (yes/no) (post operation)	0.679	0.433–1.065	0.092						
AQP1 expression (high/low)	1.954	1.185–3.222	0.009	1.449	0.858–2.448	0.166			
β-catenin expression (high/low)	1.805	1.059–3.075	0.030				1.523	0.879–2.637	0.133

Recently, β-catenin was demonstrated to be involved in AQP1-mediated cell migration [15–17]. As a water channel protein of epithelial and endothelial cells, AQP1 is reported to be also associated with cells proliferation, migration and invasion in various malignancies by current studies [19–21]. However, the role of AQP1 in astrocytoma progression has not been systematically analyzed and no any clinical evidence was provided about the association of AQP1 and β-catenin until present. Therefore, we focused on AQP1 and its relationship with β-catenin in the following studies.

**Figure 2:**
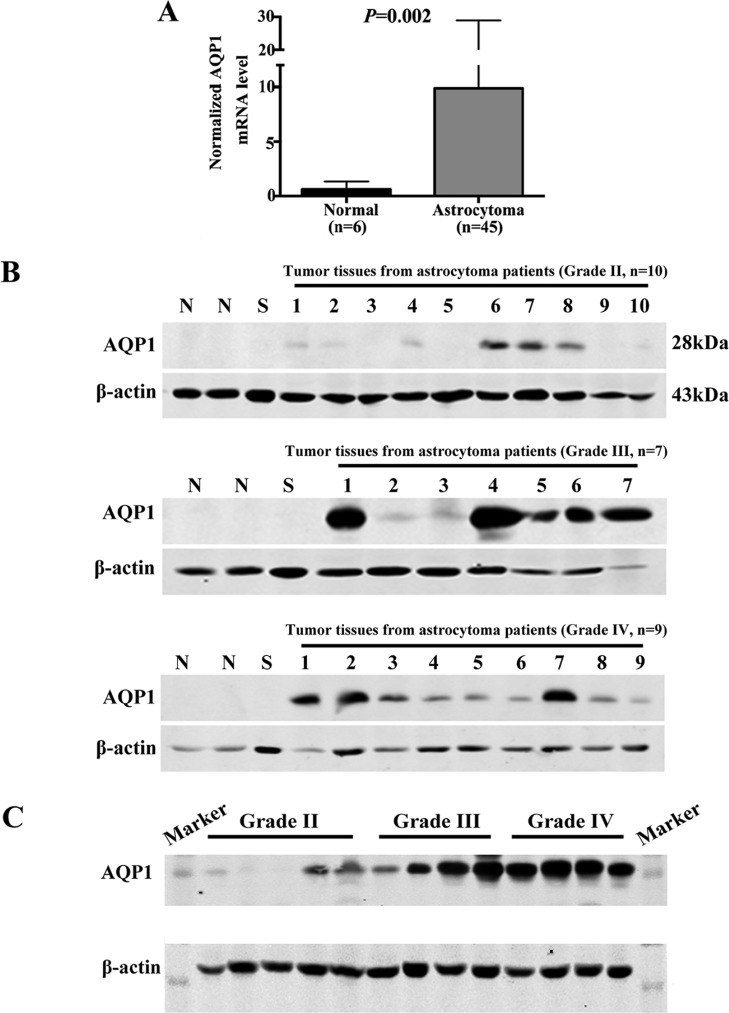
Expression of AQP1 was up-regulated in astrocytoma tissues (**A**) Normalized AQP1 mRNA levels were analyzed based on gene expression profiling data from ONCOMINE (www.oncomine.org) database, including 45 astrocytoma tissues and 6 normal temporal lobe samples. (**B**) Western blot analysis of AQP1 protein expression in human astrocytoma tissues with different histological grades (1–10 stands for tumor tissues from 10 patients with Grade II astrocytoma, 1–7 stands for tumor tissues from 7 patients with Grade III astrocytoma, 1–9 stands for tumor tissues from 9 patients with Grade II astrocytoma). N (Non-neoplastic tissues adjacent to tumor) and S (Schwannoma) were regarded as controls. (**C**) Western blot result of AQP1 protein expression in astrocytoma specimens with different pathological grades in one gel (Grade II: 5 cases; Grade III: 4 cases; Grade IV: 4 cases). β-actin was used as a loading control.

We first analyzed AQP1 mRNA expression level in gene expression profiling data (mined from the publicly available ONCOMINE database, www.oncomine.org), including 45 astrocytoma cases and 6 normal temporal lobe samples. The validation data confirmed that it was up-regulated in astrocytoma compared with normal tissues (*P* = 0.002, Figure 2A). Then western blot analysis of AQP1 protein expression was performed using fresh frozen astrocytoma specimens. 2 non-neoplastic tissues adjacent to tumor and 1 Schwannoma sample were used as controls. Our results showed that AQP1 expression was too low to be detected in the control group, whereas it exhibited higher expression in both Grade III and Grade IV than Grade II astrocytoma specimens (Figure 2B–2C).

**Figure 3:**
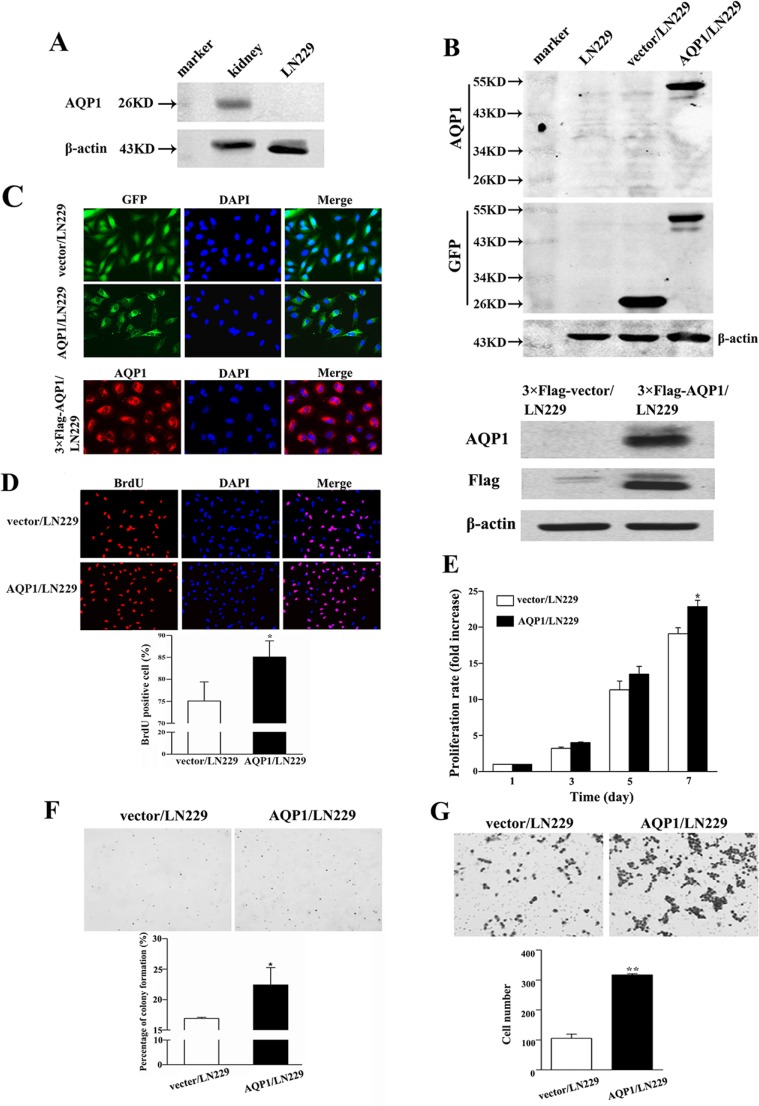
Overexpression of AQP1 promoted proliferation and invasion abilities of LN229 cells (**A**) In parental LN229 cells, AQP1 expression was too low to be detected by western blot. Mouse kidney tissue was used as a positive control. (**B**) Establishment of stable AQP1-overexpressing cells through lentiviral transfection of GFP labeled AQP1-expressing vector (upper part) and 3×Flag labeled AQP1-expressing vector (lower part). (**C**) Exogenous AQP1 expression was observed in AQP1/LN229 (upper part) and 3×Flag-AQP1/LN229 cells (lower part) by GFP fluorescence or anti-AQP1 antibody, respectively (200×). (**D**) Proliferation ability was detected by BrdU incorporation analysis in AQP1/LN229 and control cells (200×). (**E**) Proliferation assay was performed by MTT assays in AQP1/LN229 and control cells. (**F**) Colony-forming ability was examined in AQP1/LN229 and control cells (200×). (**G**) Invasion assays were performed in AQP1/LN229 and control cells (200×). All experiments were performed 3 times independently (Student’s *t*-test, ^*^P < 0.05, ^**^P < 0.01).

In the following studies, we conducted *in vitro* experiments to validate the function of AQP1 in astrocytoma. Since endogenous AQP1 was undetectable in parental LN229 cells, we over-expressed GFP-AQP1 fusion protein and 3×Flag-AQP1 fusion protein in LN229 cells (designed as AQP1/LN229 and 3×Flag-AQP1/LN229, respectively). The exogenous expression of AQP1 was confirmed by Western blot and kidney tissue from mouse was used as a positive control (Figure 3A and 3B). As shown in Figure 3C, exogenous expression of AQP1 was predominantly localized in cytoplasm of both AQP1/LN229 and 3×Flag-AQP1/LN229 cells. AQP1 overexpression increased cells proliferation of LN229 cells, validated by BrdU incorporation assays, MTT assays and Soft agar assays, respectively (Figure 3D–3F). Furthermore, increased invasion was observed in AQP1/LN229 cells compared with control (Figure 3G).

**Figure 4:**
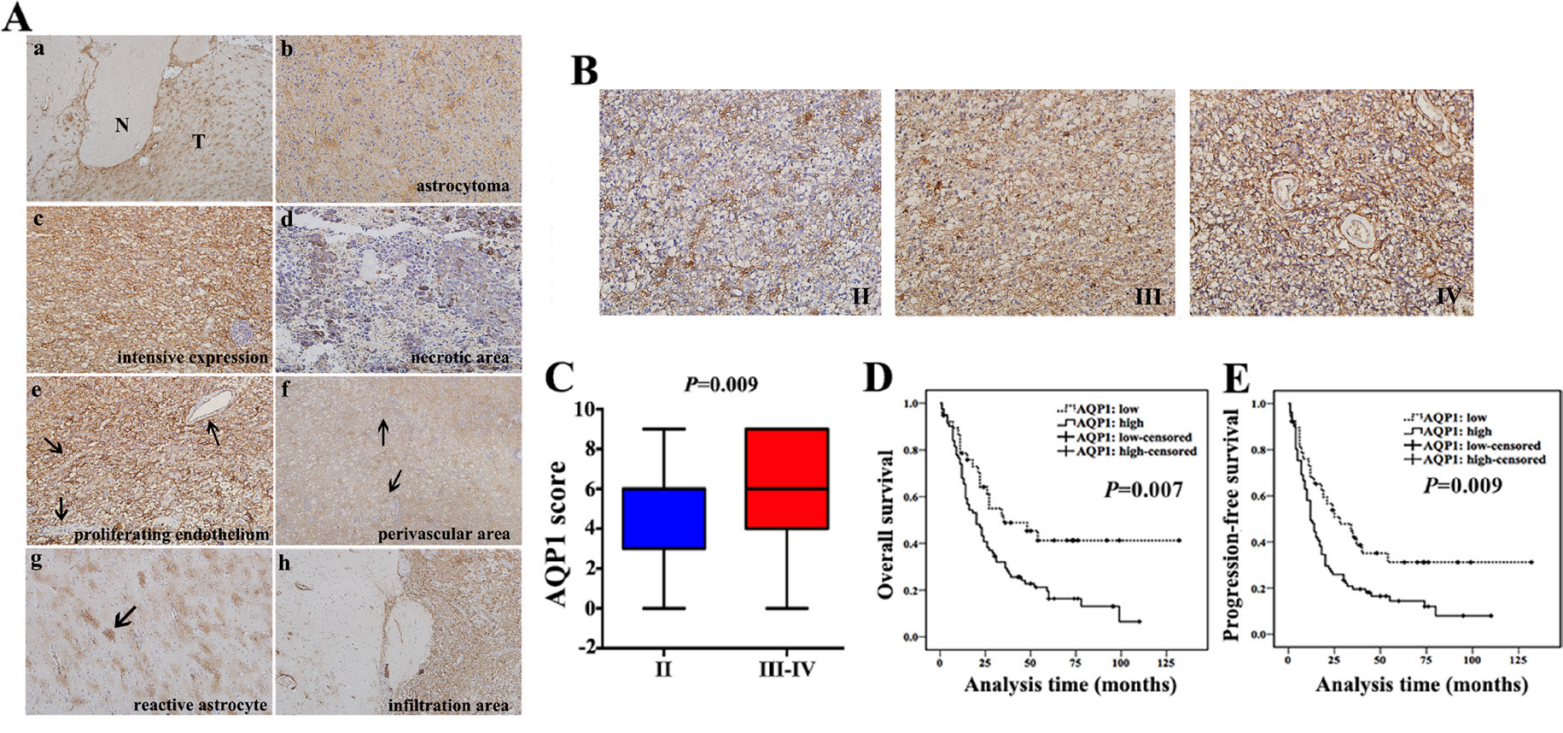
AQP1 expression increased with histological grade and inversely correlated with patients’ outcome (**A**) Expression pattern of AQP1 in astrocytoma tissues. (a) Astrocytoma tissue including tumor regions (T) and normal brain regions (N) (40×). (b) AQP1 expression presented a typical astrocytic appearance (200×). Grade IV astrocytoma exhibited intensive AQP1 expression in tumor cells (c), with a lack or weak expression close to necrotic areas (d) (200×). (e) AQP1 was absent in the proliferating endothelial cells in Grade IV astrocytoma (200×). (f) Clustered AQP1 expression around vessels was observed in Grade IV astrocytoma (40×). (g) AQP1 expression was observed in reactive astrocytes (100×). (h) AQP1 was expressed in tumor infiltration areas (100×). (**B**) Representative images of AQP1 expression in Grade II, III and IV of astrocytoma (200×). (**C**) Ranges and median values of AQP1 score in astrocytoma specimens AQP1 score of Grade II was median: 6, range: 0–9, mean: 4.6, mean rank: 66.4. AQP1 score of Grade III–IV was median: 6, range: 0–9, mean: 5.9, mean rank: 86.4. (Mann-Whitney U test, *P* = 0.009). (**D** and **E**) High expression of AQP1 indicated a shorter OS (D) and PFS (E) in astrocytoma patients (log-rank test).

Next, we investigated clinical role of AQP1 by the same cohort of 160 astrocytoma specimens. AQP1 expression was not observed in glial cells from normal brain regions, whereas it was up-regulated in tumor regions (Figure 4Aa) and it exhibited a typical astrocytic appearance (Figure 4Ab). In certain areas, AQP1 was consistently expressed in tumor cells and displayed no association with specific structures (Figure 4Ac). However, in other areas, deficient/weak AQP1 expression was observed around necrotic sectors (Figure 4Ad). AQP1 expression was absent in the proliferating endothelium of Grade IV astrocytoma (Figure 4Ae), whereas in perivascular areas, it exhibited a more clustered and denser distribution (Figure 4Af). Moreover, AQP1 could also be expressed in reactive astrocytes (Figure 4Ag) and the areas of tumor infiltration (Figure 4Ah). As shown in Figure 4B, AQP1 expression was predominantly confined to the membrane of a proportion of tumor cells in Grade II astrocytoma, while there was wider and denser membranous and intracytoplasmic expression in Grade III and Grade IV and AQP1 expression was higher in high-grade than of low-grade group (*P* = 0.009, Figure 4C). Expression of AQP1 was also positively correlated with histological grade (*r*_s_ = 0.163, *P* = 0.039, Table 3) and high expression of AQP1 indicated worse outcome of astrocytoma patients (Figure 4D and 4E). AQP1 was not an independent prognostic factor in multivariate analysis stratified by age, tumor size and histological grade (*P* = 0.166, Table 4).

**Figure 5:**
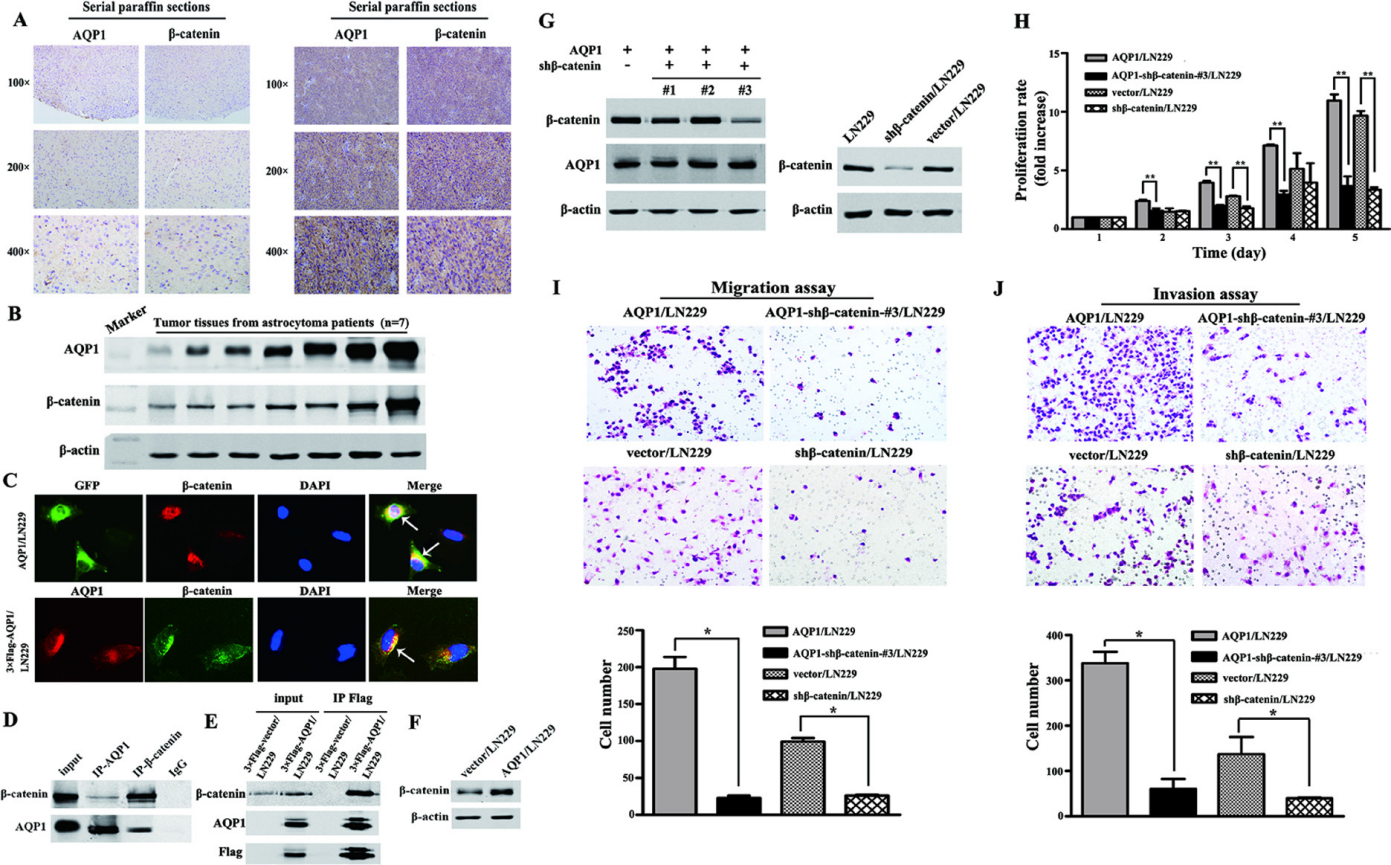
Relationship between AQP1 and β-catenin in astrocytoma (**A**) Expression of AQP1 was positively correlated with β-catenin expression. The expression of AQP1 and β-catenin were detected using serial paraffin sections by immunohistochemistry analysis. (**B**) The expression of AQP1 and β-catenin were detected by western blot using tumor tissues from astrocytoma patients (7 cases). (**C**) Co-localization of AQP1 and β-catenin in AQP1/LN229 and 3×Flag-AQP1/LN229 cells, respectively (200×). (**D**) Co-immunoprecipitation results of AQP1 and β-catenin in astrocytoma tissues. (**E**) Co-immunoprecipitation results of AQP1 and β-catenin in 3×Flag-AQP1/LN229 cells. (**F**) β-catenin was upregulated in AQP1/LN229 cells compared with control. (**G**) Knocking down β-catenin expression in LN229 and AQP1/LN229 cells (#1, #2 and #3). The vector/LN229 cell line was used as a control group. (**H**) Proliferation experiments by MTT assays. (**I**) Migration experiments results (200×). (**J**) Invasion experiments results (200×). All experiments were performed 3 times independently (Student’s *t*-test, ^*^P < 0.05, ^**^P < 0.01).

To further validate the relationship between AQP1 and β-catenin, combinatorial analysis of AQP1 and β-catenin was evaluated by the same cohort of 160 astrocytoma specimens and we demonstrated for the first time that AQP1 expression positively correlated with β-catenin (*r*_s_ = 0.158, *P* = 0.045, Table 5, Figure 5A–5B). Our immunofluorescence analysis also confirmed their co-localization in cytoplasm of AQP1/LN229 cells and 3×Flag-AQP1/LN229 cells (Figure 5C). In addition, our results revealed that AQP1 and β-catenin could co-immunoprecipitate with each other in astrocytoma tissues and 3×Flag-AQP1/LN229 cells, respectively (Figure 5D and 5E). Furthermore, overexpression of AQP1 upregulated expression of β-catenin (Figure 5F). Next, β-catenin expression was knocked down by 5 different shRNA in AQP1/LN229 cells and we found down-regulation of β-catenin in AQP1 overexpression cells suppressed cells proliferation, migration and invasion ability (Figure 5G–5J, Supplementary Figure 1).

**Table 5:** Distribution (mean, range) of AQP1, β-catenin score; the rate of high AQP1 or β-catenin expression as per histological grade in astrocytoma and the correlation between AQP1 and β-catenin

Variable	Grade II	Grade III	Grade IV	Total	*r*_s_	*P* value^a^
AQP1 (160 cases)	4.64 (0–9)	5.85 (0–9)	6.02 (0–9)	5.56 (0–9)	0.158	0.045
	28/47	32/48	48/65	108/160		
	(59.6%)	(66.7%)	(73.8%)	(67.5%)		
β-catenin (160 cases)	104.7 (0–300)	144.0 (0–300)	158.0 (0–300)	138.1 (0–300)		
	24/47	37/48	50/65	111/160		
	(51.1%)	(77.1%)	(76.9%)	(69.4%)		

^a^*P* value was calculated by Spearman’s Rank-Correlation test.

**Figure 6:**
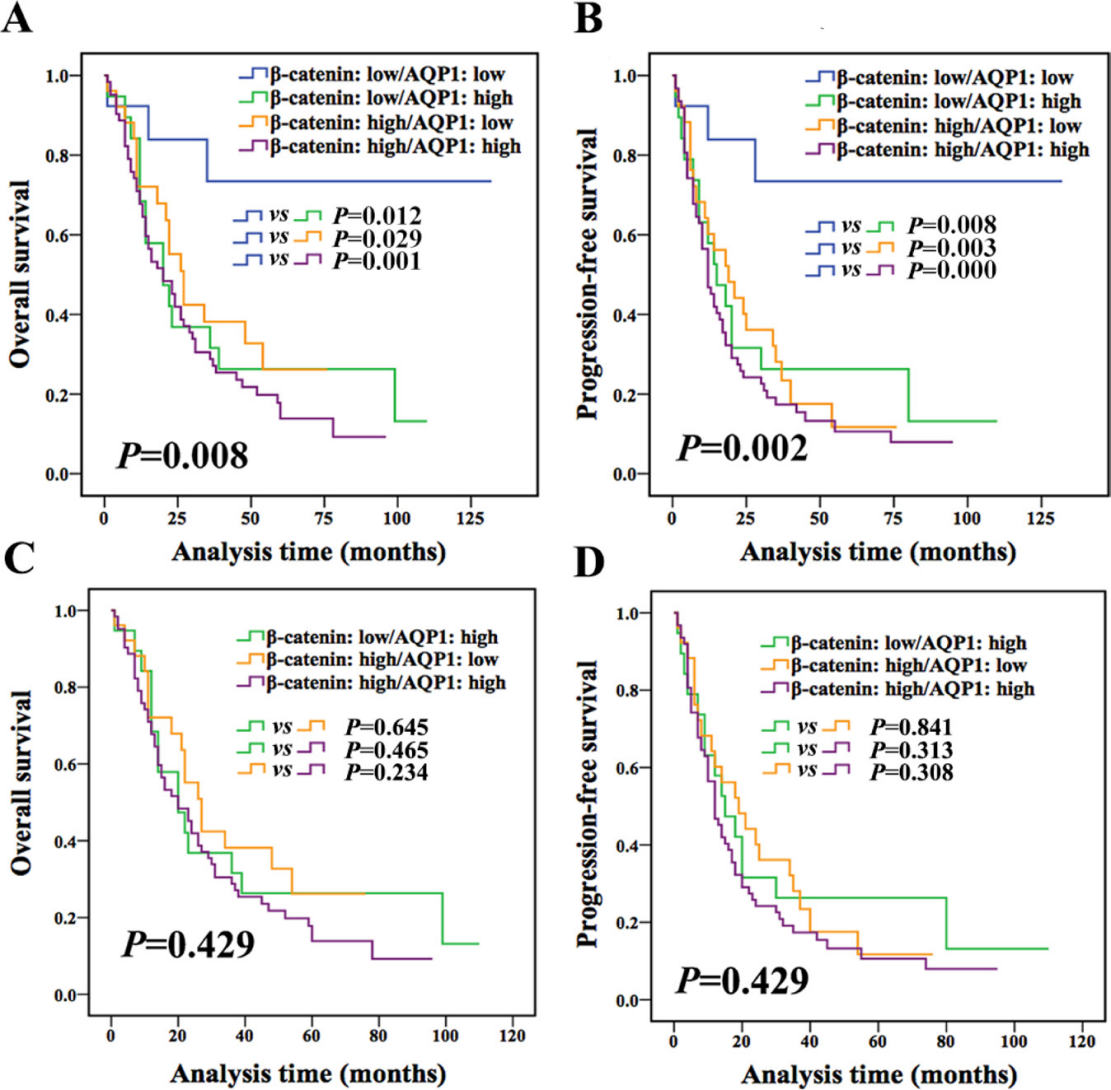
Combination of AQP1 and β-catenin provided more accurate information for prognosis prediction of astrocytoma patients (**A** and **B**) Patients were divided into four groups according to the expression of both AQP1 and β-catenin. Patients with AQP1 low/β-catenin low expression had the best outcome compared with the other three groups (log-rank test). (**C** and **D**) No statistical difference was observed between any two groups among the other 3 groups (AQP1 low/β-catenin high, AQP1 high/β-catenin low, AQP1 high/β-catenin high, log-rank test).

In the following, according to expression of both AQP1 and β-catenin, patients were divided into 4 groups (AQP1 low/β-catenin low, AQP1 low/β-catenin high, AQP1 high/β-catenin low, and AQP1 high/β-catenin high). Patients with AQP1 low/β-catenin low exhibited better outcome compared with other 3 groups (Figure 6A–6B), while other 3 groups had a similar prognosis (OS: *P* = 0.429, PFS: *P* = 0.429, Figure 6C and 6D). No statistical difference was observed between any two groups among the other 3 groups (Figure 6C and 6D). Based on above analysis, we concluded that patients with low expression of both AQP1 and β-catenin had the best prognosis, while patients with either high expression of AQP1 or β-catenin or both of them showed worse prognosis.

Therefore, combination of AQP1 and β-catenin expression had a better prognostic value and it was further confirmed by multivariate analysis. As shown in Table 6 and Table 7, both univariate and multivariate Cox proportional hazard regression analysis demonstrated that combination of AQP1 and β-catenin expression was an independent factor for astrocytoma (OS: *P* = 0.048; PFS: *P* = 0.015) independent of age, tumor size and histological grade.

**Table 6:** Univariate and multivariate analysis for AQP1/β-catenin expression in overall survival (OS) analysis

Variables	Univariate			Multivariate		
	HR	95% CI	*P* value	HR	95% CI	*P* value
Age	1.031	1.017–1.045	0.000	1.025	1.010–1.041	0.001
Gender (male/female)	0.949	0.620–1.455	0.812			
Tumor size (≤ 5 cm versus > 5 cm)	1.795	1.169–2.758	0.008	1.961	1.247–3.084	0.004
Histological grade (II versus III versus IV)	1.966	1.486–2.599	0.000	1.687	1.253–2.272	0.001
Peritumoral edema	1.249	0.951–1.641	0.110			
Surgical excision (complete versus partial)	0.697	0.399–1.216	0.204			
Radiotherapy (yes/no) (post operation)	0.725	0.476–1.105	0.135			
Chemotherapy (yes/no) (post operation)	0.679	0.433–1.065	0.092			
AQP1/β-catenin expression	1.407	1.121–1.765	0.003	1.280	1.002–1.634	0.048
AQP1 low/β-catenin low						
AQP1 low/β-catenin high						
AQP1 high/β-catenin low						
AQP1 high/β-catenin high						

**Table 7:** Univariate and multivariate analysis for AQP1/β-catenin expression in progression-free survival (PFS) analysis

Variables	Univariate			Multivariate		
	HR	95% CI	*P* value	HR	95% CI	*P* value
Age	1.032	1.018–1.046	0.000	1.026	1.011–1.041	0.001
Gender (male/female)	0.939	0.622–1.417	0.763			
Tumor size (≤ 5 cm versus > 5 cm)	1.606	1.057–2.441	0.027	1.719	1.104–2.677	0.017
Histological grade (II versus III versus IV)	2.023	1.547–2.647	0.000	1.739	1.310–2.309	0.000
Peritumoral edema	1.372	1.051–1.792	0.020			
Surgical excision (complete versus partial)	0.820	0.490–1.372	0.451			
Radiotherapy (yes/no) (post operation)	0.750	0.499–1.126	0.165			
Chemotherapy (yes/no) (post operation)	0.738	0.480–1.137	0.168			
AQP1/β-catenin expression	1.460	1.174–1.816	0.001	1.334	1.058–1.682	0.015
AQP1 low/β-catenin low						
AQP1 low/β-catenin high						
AQP1 high/β-catenin low						
AQP1 high/β-catenin high						

## DISCUSSION

In our study, endogenous AQP1 expression could not be detected in LN229 cell line by Western blot. Only endogenous AQP1 expression in astrocytoma tissues could be found by immunohistochemistry analysis and Western blot. Previous study have pointed out that glioma cells were isolated and grown as cell lines would lose AQP1 expression such as U87 (astrocytoma, WHO grade III), STTG-1 (anaplastic astrocytoma, WHO grade III) and D54 (WHO grade IV) [22]. This loss of AQP1 in long-term tumor cell cultures and cell lines is likely a result of culture condition.

In the present study, children patients were included in the cohort. According to WHO Classification of Tumors of the Central Nervous System 2016 (Revised 4th edition) and related literature, for diffuse midline glioma, H3 K27-mutant diffuse midline glioma predominates in children but can also be seen in adults. The finding of H3 K27-matation confers a worse prognosis than that of wildtype cases [23, 24]. Therefore, classsifying glioma population based on the biomarkers beside of traditional histological grade will be more precise.

Accumulation of cytoplasmic β-catenin is an indispensable step for its translocation to nucleus [25]. In this study, aberrant cytoplasmic accumulation of β-catenin was found in majority of astrocytoma patients. We found that cytoplasmic expression of β-catenin increased with pathological grades and high expression of β-catenin indicated worse survival. These findings provided further evidence that decreased levels of β-catenin at the cell membrane as well as aberrant accumulation in the cytoplasm/nucleus might be one of the leading causes of tumor progression in astrocytoma patients. In addition, it has been known that when Wnt ligand is absent, cytoplasmic β-catenin will be phosphorylated which allows it to be recognized for proteasomal degradation. In the presence of Wnt ligand, β-catenin will be allowed to transport to the nucleus where it serves as a co-activator for TCF to activate Wnt responsive genes [26]. Previous study reported that treatment of AQP1-silenced cells with the proteasome inhibitor MG132 induced the recovery of β-catenin [15]. Based on above, we speculated that AQP1 could negate proteasomal degradation of β-catenin facilitating β-catenin into nucleus and then contributed to cell proliferation and invasion. Our recent study demonstrated that overexpression of AQP1 in tumor cells could promote proliferation and invasion ability and high cytoplasmic expression of AQP1 indicated worse outcome of breast cancer patients [27]. Here, we got similar observations in glioma cells and astrocytoma patients.

Altered expression and/or sub-localization of β-catenin have been reported to be correlated with poor survival in patients with craniopharyngioma, colorectal cancer, and breast carcinoma [28–30]. However, until present, no convincing report on the prognostic significance of β-catenin in astrocytoma is provided. Zhang *et al.* reported that the prognosis of patients with low expression of β-catenin tended to be better compared with patients with high expression of β-catenin by detecting 63 samples of astrocytoma [31]. While in another research using 81 cases of astrocytoma, β-catenin expression was not related to patients’ survival [10]. In this study, we confirmed that β-catenin alone was not an independent prognostic marker for astrocytoma using a large cohort of 160 astrocytoma specimens. Most importantly, we demonstrated for the first time that combined AQP1 and β-catenin expression was an independent prognosis factor for both OS and PFS of astrocytoma. AQP1 expression combined with β-catenin could be used to re-classify astrocytoma patients into four prognostic groups and patients with AQP1 low/β-catenin low expression had better outcome than others (with either high expression of AQP1 or β-catenin: AQP1 low/β-catenin high, AQP1 high/β-catenin low, and AQP1 high/β-catenin high).

In conclusion, combined AQP1 and β-catenin expression may be used as a powerful predictor of survival of astrocytoma patients. It showed greater prognostic value than single marker and could be more reliable for clinical application.

## MATERIALS AND METHODS

### Ethical statement

This study was reviewed and approved by Institutional Ethics Committee of Tianjin Medical University Cancer Institute & Hospital, and all the patients signed an informed consent for participation of the study and the use of their biological tissues prior to surgery. All experiments were performed in accordance with relevant guidelines and regulations of Institutional Ethics Committee of Tianjin Medical University Cancer Institute & Hospital.

### Patient selection and clinical information

Paraffin-embedded specimens from 160 astrocytoma patients diagnosed between 1999 and 2013 were randomly selected. All patients had undergone surgical resection at the Tianjin Medical University Cancer Institute & Hospital and no patients had received radiotherapy or chemotherapy prior to resection. Two pathologists independently reviewed all histological slides and graded each astrocytoma according to 2007 World Health Organization criteria [32]. Patients’ clinicopathological information was shown in Table 1. Postoperative radiation consisted of a total dose of 60 Gy in 30–33 fractions. Temozolomide was administered in postoperative chemotherapy. Prognostic information was available for 120 patients, who were followed up until September 2015 (1–132 months). During this period, 94 patients suffered recurrence and 88 patients died of tumor. Survival time was calculated from the date of surgery to astrocytoma-related death or systemic relapse, or censored at the time of the last follow-up visit or at non-cancer-related death.

### Immunohistochemistry (IHC) analysis and evaluation

In brief, sections (5 μm thick) were dewaxed, hydrated, and heated for antigen retrieval. They were blocked with hydrogen peroxide and normal goat serum, and subsequently incubated overnight with rabbit anti-human polyclonal antibody against AQP1 (1:500, SC-20810, Santa Cruz, USA) or mouse anti-human polyclonal antibody against β-catenin (1:150, SC-7963, Santa Cruz, USA). All sections were stained with 3,3′-diaminobenzidinetetra-hydrochloride (DAB).

Stained tissue sections were reviewed by two pathologists in a blinded manner based on a double scoring system (staining intensity multiplied by staining area). Staining intensity for AQP1 and β-catenin was scored as follows (0: no staining; 1: definite but weak staining; 2: moderate staining; 3: strong staining). The positive staining area of AQP1 was scored as follows: 0 (0%–25%); 1 (26%–50%); 2 (51%–75%); 3 (76%–100%), producing a total scores range of 0 to 9. The positive staining area of β-catenin was scored as 0–100, producing a total score range of 0 to 300. In our present study, low and high expression of AQP1 was regarded as score 0–4 and score 5–9, respectively. Low and high expression of β-catenin was regarded as score 0–99 and score 100–300, respectively.

### Construction of lentiviral vector expressed with full length of AQP1

LN229 human glioblastoma cell line was obtained from the American Type Culture Collection (Manassas, VA, USA) and cultured in RPMI-1640 medium supplemented with 10% fetal bovine serum in a 5% CO_2_ incubator at 37°C. Cells had been tested and authenticated by DNA (STR) profiling, work performed by Beijing Microread Genetics Co., Ltd. (Beijing, China).

Full length of AQP1 was amplified by PCR using primers for human AQP1 (GenBank accession No. NM_198098.2, Forward: 5′-AATTGAATTCGCCACCATGGCCAGCGAGTTCAAG-3′ and Reverse: 5′-CGGGATCCCTATTTGGGCTTCATCTC-3′). AQP1 with a green fluorescent protein (GFP) label or 3×Flag label was cloned into pCDH-CMV-MCS-EF1-Puro lentiviral vector (http://www.addgene.org/). The sequences of the inserts were 100% correct. Lentiviruses were produced by co-transfection of lentiviral plasmid, packing plasmids ΔR and pVSVg into HEK-293T cells. After transfection, supernatant was collected and virus was used to infect LN229 cells. Lentivirus-infected cells were screened by 1 μg/ml puromycin for 2 weeks to establish stably cell clones (named AQP1/LN229) and verified by western blot analysis. GFP-labeled AQP1-overexpressed cells were named AQP1/LN229 and 3×Flag labeled AQP1-overexpressed cells were named 3×Flag-AQP1/LN229, respectively.

### β-catenin knockdown in AQP1-overexpressed cells

5 different β-catenin specific RNA interference sequences were applied (#1-#5). The sequences were following: (#1: 5′-gctccttctctgagtggtaaa-3′); (#2: 5′-aggtgctatctgtctgctcta-3′); (#3: 5′-ttgttatcagaggactaaata-3′); (#4: 5′-gttctccgaacgtgtcacgt-3′); (#5: 5′-gcttggaatgagactgctgat-3′). Lentiviruses were produced by co-transfection of lentiviral vector and packing plasmids ΔR and pVSVg into HEK-293T cells. Supernatant was collected and virus was used to infect cultured AQP1 over-expressing LN229 cells. Expression of β-catenin was verified by western blot analysis and these cells were named AQP1-shβ-catenin/LN229.

### Immunofluorescence analyses

Cells were seeded in 35-mm dishes 24 hours before they were fixed. In AQP1/LN229 cells, AQP1 localization was directly analyzed by GFP fluorescence. In 3×Flag-AQP1/LN229 cells, AQP1 was stained with Alexa fluor 546-conjugated goat anti-rabbit IgG (A11010, Life technologies) after incubated with primary AQP1 antibody at 4°C overnight. β-catenin was stained with Alexa fluor 594-conjugated goat anti-mouse IgG (A11005, Life technologies) in AQP1/LN229 cells and Alexa fluor 488-conjugated goat anti-mouse IgG (A11001, Life technologies) in 3×Flag-AQP1/LN229 cells after incubated with primary β-catenin antibody at 4°C overnight. DAPI (4′, 6-diamidino-2-phenylindole) was used to stain nuclei. Images were acquired using a fluorescence microscope (magnifications 200×).

### Co-immunoprecipitation (Co-IP) and Western blot

Astrocytoma tissues were washed three times with ice-cold PBS and then re-suspended with CO-IP lysis buffer (pH 7.4). Tissue lysates were gently rotated at 4°C overnight followed by centrifuging at 12,000g for 5 minutes and the pellet was discarded. Nonspecific protein was removed by adding Protein A (SC-2001, Santa Cruz, USA) and gently rotated at 4°C for 1 hour. Then the mixture was centrifuged and the supernatant was divided into three groups with following different antibodies treatments. The precipitates were washed three times with CO-IP lysis buffer, re-suspended in sample buffer and boiled for 5 minutes for western blot examination.

Cells (3×Flag-AQP1/LN229 and 3×Flag-vector/LN229) were washed three times with ice-cold PBS and then re-suspended with CO-IP lysis buffer (pH 7.4). Cell lysates were gently rotated at 4°C overnight followed by centrifuging at 12,000g for 10 minutes and the pellet was discarded. The supernatant was immunopurified with anti-Flag M2 affinity gel (A2220, Sigma) and eluted with Flag peptides. The eluates were re-suspended in sample buffer and boiled for 5 minutes for Western blot examination.

Frozen astrocytoma specimens of Figure 2 and Figure 5B were collected between 2010 and 2013. All tumor tissues in the present study were obtained from primary resections, and no patients had undergone radiotherapy or chemotherapy prior to resection. Control brain specimens were obtained from 2 non-neoplastic tissues adjacent to tumor and 1 Schwannoma patient. Tissues or cells were lysed in 1×SDS lysis buffer (Tris-HCl, pH 6.8, 62.5 mM, 2% SDS, 10% glycerol) and equal amounts of protein were separated by SDS-PAGE. The blots were incubated by a primary antibody: anti-AQP1 (1:500), β-catenin (1:2000), GFP (1:7000, KM8009, SanJian, China), Flag (1:8000, AF519-1, Beyotime, China) and β-actin (1:5000, SC-47778, Santa Cruzm USA). The membrane was treated with secondary antibodies and blots were analyzed using LiCor Odyssey infrared imaging.

### BrdU labeling

Cells were seeded in 35-mm dishes and cultured for 72 hours. Following treatment with BrdU (5-bromo-2′-deoxyuridine, 1 mg/ml) for 48 hours, cells were incubated with mouse anti-BrdU antibody (1:200, ZM0013, ZhongShan, China), followed by incubation with Alexa Fluor 594-conjugated secondary antibody (goat anti-mouse, 1:100, ZF-0513, ZhongShan, China) for 1 hour in the dark. Finally, cells were stained with DAPI (1 µg/ml) and BrdU-DAPI double-positive cells were regarded as BrdU-positive cells.

### MTT assay

Cells were plated and incubated in 24-well plates. At the end of the incubation period, viable cells were quantified using 3-(4, 5-dime- thylthiazol-2-yl)-2, 5-diphenyltetrazolium bromide (MTT). Briefly, 50 μl of the MTT stock solution (5 mg/ml) was added to each well. After 4 hours of incubation, medium was removed and the converted dye was solubilized with dimethyl sulfoxide. The absorbance of the converted dye was measured at a wave length of 570 nm.

### Soft agar assays

Briefly, stably transfected cells (AQP1/LN229 or vector/LN229) were seeded in complete medium in six-well culture plates containing a top layer of 0.35% agar and a bottom layer of 0.6% agar. The plates were then incubated at 37°C for 4 weeks and later stained with 0.005% crystal violet for 1 hour. Colonies larger than 50 µm were scored and photographed at 200× magnifications using an Olympus microscope (Olympus, Japan).

### Migration assay

Migration assays were performed using 24-well transwell migration chambers (Corning, New York, USA) with polyethylene membranes (8 μm pore size). The upper chambers were seeded with 1.0×10^5^ cells/well in 200 μl of serum-free DMEM containing 0.1% bovine serum albumin (BSA). 600 μl medium (serum-free DMEM containing 0.1% BSA) with 50 ng/ml recombinant human epithelial growth factor (EGF, Minneapolis, MN, USA) were added to the lower chambers. The cells were allowed to migrate for 24 hours at 37°C. Afterward, cells at the upper layer of the membrane were scraped and cells at the lower layer were stained with Giemsa solution and photographed under a microscope. The number of cells was quantified in randomly selected fields.

### Matrigel invasion assay

Boyden chamber invasion assays were performed to measure cell invasion *in vitro*. Briefly, transwell inserts for 24-well plates were coated with prediluted matrigel. 1.0 × 10^5^ cells/well were loaded into the upper compartment of the chambers, and medium (serum-free DMEM containing 0.1% BSA) with 50 ng/ml EGF was added to the lower chambers. After 24 hours of incubation, the invading cells were fixed, stained, counted and photographed under a microscope in five pre-determined fields at 200× magnifications.

### Statistical analyses

The SPSS 17.0 software package was used for statistical analysis. Mann-Whitney U test, ANOVA test and χ^2^ test were performed for group comparisons and correlations between two variables were evaluated by Spearman’s Rank-Correlation test. Overall survival (OS) and progression-free survival (PFS) rates were estimated using the Kaplan-Meier method, and the log-rank test was applied to compute *P*-values. The Cox proportional hazards regression model was performed toward the identification of relevant prognostic factors. For analysis of *in vitro* cellular experiments, statistical significance for comparisons between groups was determined using a two-tailed Student’s *t*-test. All data was presented as mean ± standard deviation. Three independent experiments were performed. A two-sided *P*<0.05 was considered statistically significant in all analyses.

## SUPPLEMENTARY MATERIALS FIGURE


