# Supplementary material for: Combination of AQP1 and β-catenin expression is an independent prognosis factor in astrocytoma patients
Source: Oncotarget. 2017 Jul 26;8(59):99414–28. doi: 10.18632/oncotarget.19562 (PMC5725103; doi:10.18632/oncotarget.19562)
Supplement: Supplementary file 1 [file oncotarget-08-99414-s001.pdf]

Combination of AQP1 and β-catenin expression is an independent prognosis factor in astrocytoma patients

SUPPLEMENTARY MATERIALS

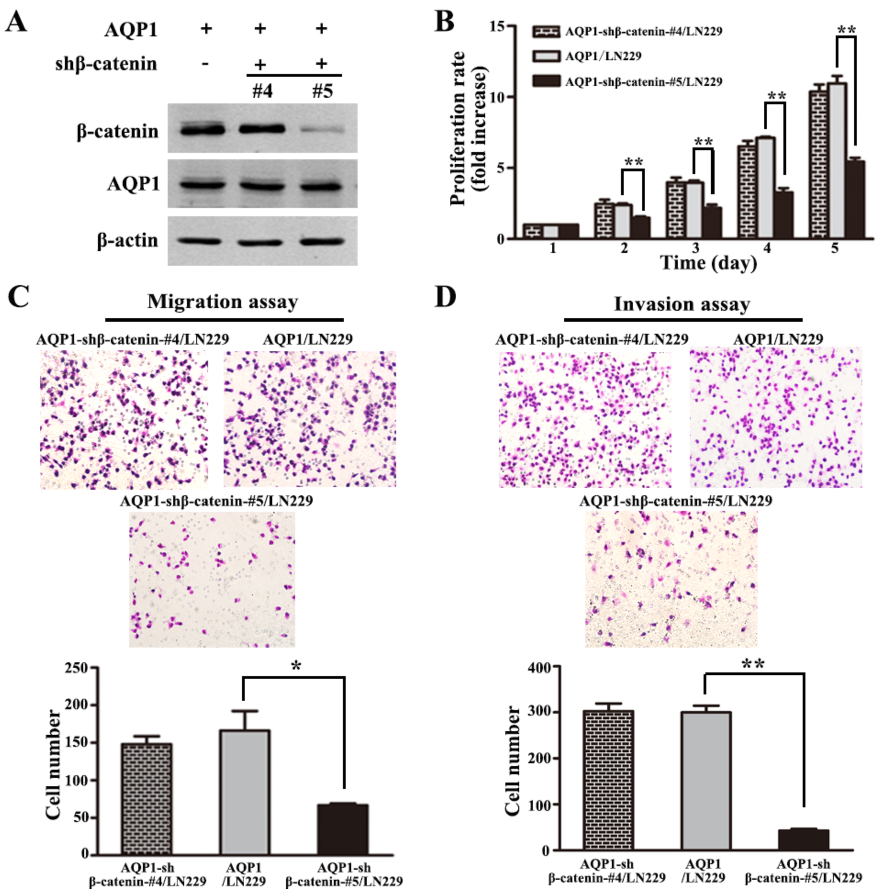

Supplementary Figure 1: Down-regulation of β-catenin decreased the proliferation, migration and invasion of AQP1/LN229 cells. (**A**) β-catenin expression was knocked down in AQP1/LN229 cells and 2 different RNA interference sequences were applied (#4 and #5). Expression level of β-catenin and AQP1 was detected by Western blot. (**B**) Down-regulation of β-catenin decreased proliferation of AQP1/LN229 cells. (**C**) Down-regulation of β-catenin inhibited migration of AQP1/LN229 cells (200×). (**D**) Down-regulation of β-catenin inhibited invasion abilities of AQP1/LN229 cells (200×). All experiments were performed 3 times independently (Student's *t*-test).
